# The Synergism of 1-Methylcyclopropene and Ethephon Preserves Quality of “Laiyang” Pears With Recovery of Aroma Formation After Long-Term Cold Storage

**DOI:** 10.3389/fpls.2020.00490

**Published:** 2020-05-25

**Authors:** Pan Shu, Dedong Min, Jingxiang Zhou, Wen Ai, Jiaozhuo Li, Zilong Li, Xinhua Zhang, Zedong Shi, Yingjie Sun, Fujun Li, Xiaoan Li, Yanyin Guo

**Affiliations:** School of Agricultural Engineering and Food Science, Shandong University of Technology, Zibo, China

**Keywords:** aroma, pear (*Pyrus bretschneideri* Reld), aroma recovery, ethephon, 1-methylcyclopropene, cold storage

## Abstract

A “Laiyang” pear is a climacteric fruit with a special taste and nutritional value but is prone to a post-harvest aroma compound loss and a loss in fruit quality. In this study, pears were pretreated with 0.5 μl L^–1^ 1-methylcyclopropene (1-MCP) at 20°C for 12 h and then stored at 0 ± 1°C for 150 days to evaluate the influence of 1-MCP on fruit quality and the changes in components of volatile aromas. In addition, pears were further treated with 2 mmol L^–1^ ethephon. The effects of ethephon on the recovery of aroma production were investigated during the 150 day storage at 0 ± 1°C and the subsequent 7 day shelf life at 20 ± 1°C. Treatment with 1-MCP inhibited firmness loss, increased electrical conductivity, reduced respiration and ethylene production rates as well as the contents of soluble solids, and maintained the storage quality of the fruits. However, 1-MCP treatment inhibited the emission of volatile aromas in pear fruits by decreasing the activities of various enzymes, such as lipoxygenase (LOX), hydroperoxide lyase (HPL), alcohol dehydrogenase (ADH), pyruvate carboxylase (PDC), and alcohol acetyltransferase (AAT). During the shelf-life, activities of the above mentioned enzymes were significantly enhanced, and a higher content of volatile aromas were found in fruits treated with 1-MCP + ethephon, while other qualities were not compromised. These results showed that 1-MCP treatment could effectively maintain the quality of the “Laiyang” pear during cold storage, and the additional application of ethephon on fruits during shelf-life may be a promising way to restore volatile aromas in pear fruits after long-term storage.

## Introduction

“Laiyang” pears (*Pyrus bretschneideri* Reld), important fruits produced on a commercial scale in China for many years, are favorable for their special flavor, nutritional and medicinal functions (Liu et al., 2013; Fan et al., 2016). However, physiological disorders easily occur in this fruit at room temperature no more than 1 month after harvest, resulting in a significant loss of aroma compounds and quality deterioration, leading to great economical losses during post-harvest storage periods (Liu et al., 2013). Therefore, how to effectively control the quality deterioration and to prolong the fruit’s post-harvest life becomes a critical factor affecting the future development of this fruit.

The “Laiyang” pear has been recognized as a climacteric fruit since its ripening process is accompanied by ethylene and a respiration burst (Nath and Panwar, 2018). Methylcyclopropene (1-MCP), a strong inhibitor of ethylene perception, can effectively prevent ethylene biosynthesis, reduce tissue browning, and delay ripening of fruits and vegetables by permanently binding to ethylene receptors. 1-MCP has been successfully applied in commercial post-harvest operations to extend the storage life of climacteric and non-climacteric fruit (Blankenship and Dole, 2003; Li F. et al., 2013, Li L. et al., 2016; Liu et al., 2018). In “Huangguan” pears, the application of 1-MCP decreased the production of ethylene and respiration rates, and reduced the browning index (Xu et al., 2020). The positive role of 1-MCP had also been confirmed in the “Yali” pear. The pear fruit treated with 1.0 μl L^–1^ 1-MCP retained a higher firmness, titratable acidity (TA), and lower core browning (Cheng et al., 2019). Although, the positive roles of 1-MCP in maintaining a fruit’s physiology have been well demonstrated in various fruits, the negative effects on the formation of aroma cannot be ignored. In “d” Anjou’ pears, 1-MCP treatment decreased the prevalence of scald and internal disintegration but interfered with the “d” Anjou’ pear’s ability to ripen normally after storage (Argenta et al., 2003; Xie et al., 2014). In addition, the treatment with 1-MCP at the early ripening stage of the pear fruit, caused a failure in ripening so that the fruit did not attain the desirable taste and flavor, and did not develop a buttery texture (Moya-León et al., 2006; Hendges et al., 2018). In apples, 1-MCP treatment significantly inhibited and delayed the volatile production such as α-farnesene, alcohols, and esters (Yang et al., 2016). The volatile production evaluation demonstrated that 1-MCP treatment significantly suppressed the synthesis of saturated and unsaturated esters derived from ethylene-dependent fatty acid metabolism in papaya (Sundaram and Prabhakaran, 2017). Balbontin et al. (2007) reported that the development of ethylene-dependent flavor processes in fruit was blocked by 1-MCP treatment. Therefore, in spite of the aforementioned benefits, unfavorable influences in 1-MCP treatment on fruit aroma and ripening have also been reported.

The formation of aroma is closely related to fruit maturity and mainly involves three pathways: amino acid, fatty acid, and terpenoid metabolic pathways. Ethylene as a plant hormone participates in many ripening-related processes favorable for the synthesis of aromas (Saltveit, 1999). Wang et al. (2018) reported that most flavor-relevant compounds that accumulated the most in red-ripe tomato fruit were regulated by ethylene. Treatment of cold-stored kiwifruit with ethephon effectively enhanced the “tropical” and “fruit candy” aromas, while the “green” aroma was less obvious (Günther et al., 2015). In “Eureka” lemon, ethephon treatment significantly increased the volatile compounds in the fruit peel about 1-fold compared to the control (Zhang and Zhou, 2019). In apples, total volatiles as well as individual compounds, mainly associated with alcohols and esters, were significantly increased by ethylene treatment. Ethylene not only regulated the genes related to fatty acid synthesis and metabolism but also regulated the synthesis of esters by promoting alcohol acetyltransferase activity (Defilippi et al., 2005; Yang et al., 2016). The production of most esters in papaya was also dependent on ethylene, and the application of ethephon during fruit ripening promoted the production of esters (Balbontin et al., 2007). In addition, ethylene also played an important role in regulating the production of methyl-branched and aromatic volatile compounds from the catabolism of amino acids (Li Y. et al., 2016). However, exogenous application of ethylene or ethephon and excessive endogenous ethylene can easily lead to fruit over-ripening and senescence.

Recent studies have revealed that the simultaneous application of 1-MCP and ethylene at harvest has been tested as a strategy to recover ripening capacity of banana (Musa spp.) fruit (Botondi et al., 2014; Zhu et al., 2015) and the “Conference” pear (Chiriboga et al., 2011; Neuwald et al., 2015). However, there is no available information on the effects of 1-MCP and ethylene treatment on the aroma recovery of pear fruit during their shelf-life. The study aims to investigate the effect of 1-MCP treatment on the quality of the “Laiyang” pear during the cold storage period, and the effect of ethephon on the recovery of aroma formation of the “Laiyang” pear fruit during the shelf-life.

## Materials and Methods

### Fruit Material and Treatments

Pear fruits (*Pyrus bretschneideri* Reld cv. Laiyang) were harvested from homogeneous trees (400 fruits per tree) in a commercial orchard (fruit weight: 168.28 ± 9.71 g; soluble solids content: 12.26 ± 0.14% and firmness: 40.83 ± 1.89 N) in Laiyang, Shandong Province, China, and then immediately transported to the laboratory. Trees were grafted in 1995 on Duli (*Pyrus Betulaefolia* Bunge) rootstock, with a tree spacing of 6 m × 4 m. Fruit with similar sizes, free of mechanical damage, were selected as materials.

The pear fruits were randomly divided into two groups of 240 fruits each and subjected to the following treatments: (1) 1-MCP treatment: the fruits were fumigated with 0.5 μl L^–1^ 1-MCP at 20 ± 1°C for 12 h in a sealed container. (2) CK treatment: the fruit were sealed in a container at 20 ± 1°C for 12 h without 1-MCP. After treatment, all pear fruits were immediately transferred and stored at 0 ± 1°C with 90–92% relative humidity. At 1-month intervals, for a total storage time of 5 months, fruits were removed and allowed to equilibrate at 20°C overnight for measurements of ethylene production rate, respiration rate, aroma, and fruit quality immediately, or frozen in liquid nitrogen and stored at −80°C for enzyme assays. All treatments were conducted with three replications.

After 150 days of storage, 40 fruit from each treatment of each replicate were randomly selected from CK or 1-MCP treatment and divided into two portions, each containing 20 fruit for soaking with either 2 mmol L^–1^ ethephon (marked as CK + ethephon or 1-MCP + ethephon) or distilled water (which served as the control for ethephon treatment, marked as CK + H_2_O or 1-MCP + H_2_O) for 2 min. After drying, all fruits were stored at room temperature for 7 days before being measured.

### Determination of Ethylene Production Rate and Respiration Rate

The respiration rate and ethylene production were determined according to the method of Both et al. (2016). Five fruits from each treatment were sealed in a 4-L airtight container. After 1 h, 1 ml of the headspace gas was sampled from the container and injected into a gas chromatograph (Agilent 6890N, United States) to assay the ethylene production. The temperatures of the oven, injector, and detector were 110, 50, and 150°C, respectively. The flow rates for nitrogen, hydrogen, and compressed air were, respectively, 50, 50, and 400 ml min^–1^. The respiration rate was measured using a combined CO_2_/O_2_ analyzer (CheckMate 9900, PBI-Dansensor, Denmark). The result was expressed as nmol kg^–1^ s^–1^ for ethylene production rate, and as nmol kg^–1^ s^–1^ CO_2_ for respiration rate.

### Determination of Fruit Quality

Fruit firmness was measured using a destructive method on opposing sides of the equator region of 10 fruit per treatment with a handheld electrometer (GY-1, Mudan River, China), and the firmness was automatically calculated. Values were expressed as newton (N). After determination of firmness, the remaining parts were assessed for titratable acidity (TA) and soluble solids content (SSC). TA was determined using a base buret by titrating the juice with 0.01 mol L^–1^ NaOH up to pH 8.1. The results were expressed as g kg^–1^ of malic acid by fresh weight. SSC was measured by a manual refractometer (WYT-J, Chendu Optical Apparatus Co., Ltd., China) and the results were expressed as percentage (%).

Electrical conductivity (EC) was determined according to our previous studies with minor modifications (Zhang et al., 2013). Five pieces of fruit tissue from five fruits were obtained by steel core borer (10 mm diameter, 1 mm thick), rinsed three times and immersed in 20 ml distilled water at 25°C for 1 h. The initial EC (C_0_) was determined by DDS-307A conductivity meter (Leici Inc., Shanghai, China). Then, the fruit disks were boiled for 5 min, cooled to room temperature, and the total EC (C_1_) was monitored. The EC was defined as C_0_/C_1_ × 100%.

### Extraction and Determination of Aroma-Related Enzyme Activities

Lipoxygenase (LOX) activity was determined according to Lara et al. (2003) with minor modifications. Frozen tissue (2 g) was homogenized with 6 ml phosphate buffer containing 2 mM dithiothreitol (DTT) (pH 7.5), 1% (w/v) polyvinyl pyrrolidone (PVP) and 0.1% (V/V) Triton X-100. Then, the homogenate was centrifuged at 12 000 × *g* for 20 min at 4°C, and the supernatants were used for enzyme assays. The reaction mixture comprised of 1.5 ml of 0.1 M phosphate buffer saline (PBS), 400 μl substrate solution (8.6 mM linoleic acid, 0.25% (v/v) Tween-20, 10 mM NaOH), and 100 μl enzyme extraction. LOX activity was determined by measuring the absorbance at 234 nm for 60 s at 25°C. One unit of LOX activity was defined as an increasing of 0.01 at OD_234_ per minute.

Alcohol acetyltransferase (AAT) activity was determined as described by Pérez et al. (1996) with some modifications. Frozen tissue (2 g) was extracted in 3 ml extraction buffer containing 0.5 M Tris–HCl (pH 8.0), 0.1% (v/v) Triton X-100, and 1% (w/v) PVP. The homogenate was centrifuged at 12 000 × *g* for 20 min at 4°C, and the supernatants were used for the enzyme assays. The reaction solution contained 2 ml of 5 mM MgCl_2_, 150 μl of 5 mM acetyl-CoA, 50 μl of 200 mM butanol, 100 μl of 10 mM 5, 5′-dithiobis-bis-2-nitrobenzoic acid and 200 μl enzyme solution. AAT activity was determined by measuring the absorbance at 412 nm for 60 s at 25°C. One unit of AAT activity was defined as the increase in one unit of absorbance at 412 nm per minute.

Hydroperoxide lyase (HPL) activity was analyzed according to Vick (1991) with some modifications. Frozen tissue (2g) was extracted in 4 ml of extraction buffer containing 2-(N-morpholino) ethanesulfonic acid (MES), pH 6.5; 2 mM DTT, 1% (v/v) PVP. Then the homogenate was centrifuged at 12 000 × *g* for 20 min at 4°C. The reaction mixture contained 0.75 ml linoleate sodium hydroperoxide, 1.5 ml of 1.6 mM nicotinamide adenine dinucleotide (NAD), 0.1 ml adenine dinucleotide, and 0.5 ml crude enzyme extraction. The mixture was incubated at 30°C and then determined at 340 nm for 60 s. One unit of HPL activity was defined as an increase of 0.01 at OD_340_ per minute.

Alcohol dehydrogenase (ADH) and pyruvate carboxylase (PDC) activities were determined according to the method of Lara et al. (2003) with slight modifications. Frozen tissue (2 g) was homogenized with 4 ml MES buffer containing 2 mM DTT, 1% (w/v) PVP. Then the homogenate was centrifuged at 12 000 × *g* for 20 min at 4°C, and the supernatants were used for the enzyme assays. The ADH reaction mixture contained 0.8 ml of 100 mM MES-Tris (pH 6.5), 1.5 ml of 1.6 mM NAD, 0.05 ml of 80 mM acetaldehyde, and 0.1 ml enzyme extraction. The PDC reaction mixture contained 0.45 ml of 100 mM MES-Tris (pH 6.5), 0.1 ml of 5 mM thiamine pyrophosphate (TPP), 100 μl of 50 mM MgCl_2_, 50 μl of 1.6 mM NADH, ADH (1600U), 100 μl of 50 mM pyruvate, and 100 μl enzyme extraction. ADH and PDC activity was determined by measuring the absorbance at 340 nm for 2 min at 25°C. One unit of ADH and PDC activities was defined as an increase of 0.01 at OD_340_ per minute.

Protein content in the enzyme extracts was determined according to the method of Bradford (1976), using bovine serum albumin as a standard. Specific activities of the enzymes were expressed as units per milligram protein.

### Aroma Volatiles Analysis

Fresh fruit (10 g) containing peel from equator was homogenized with 3 ml saturated sodium chloride, and then transferred to a 30 ml vial. Before sealing of the vials, 2 μl of a solution of 0.82 g/l 3-non-anone solution was added as an internal standard. The aroma volatile compounds of pear fruits were determined using GC-MS (QP2010, Shimadzu, Japan) fitted with RTX-5 column (30 m × 0.25 mm × 0.25 μm, Agilent, United States) and based on solid-phase micro-extraction (SPME), according to a method of Cai et al. (2018). A fiber coated with 65 μm of polydimethylsiloxane and divinylbenzene (PDMS/DVB) (Supelco, Bellefonte, United States) was used for volatile compound extraction at 50°C for 30 min. The chromatographic conditions were set as follows: The furnace temperature was first maintained at 40°C for 2 min, increased at a rate of 4°C min^–1^ to 60°C and kept for 1 min, and then increased at a rate of 2°C min^–1^ to 150°C, and finally increased at a rate of 10°C min^–1^ to 210°C and kept for 5 min. Helium was used as carrier gas at a flow rate of 1.03 ml min^–1^. Electronic ionization was used at 70 eV. Detection was performed from 45 to 450 mass units. The GC-MS data processing was dealt with using Thermo XCALIBUR^TM^ 2.2 software. At the same time, retention indices (RI) were calculated by analyzing a series of n-alkanes (C_5_-C_24_) under the same conditions. The volatile aroma compounds in our experiment were identified by comparing their mass spectra with the National Institute for Standards and Technology (NIST) (Zhang et al., 2009) and by comparing it with RI reported in research. Results were expressed as μg g^–1^, and were used for hierarchical clustering heatmap analysis after homogenization.

### Statistical Analysis

Each treatment comprised three independent biological replicates. All data were expressed as the means ± standard deviation (SD) and were analyzed statistically, using one-way analysis of variance (ANOVA) and Ducan’s multiple range tests with SPSS 19.0 (SPSS Inc., Chicago, IL, United States). Volatile compound data from the four treatments were submitted to a principal component analysis (PCA), using the Origin 2018 software. In order to visualize the differences in volatile aroma composition, a hierarchical clustering heatmap was created with the heatmap3 R package.

## Results

### Effects of 1-MCP Treatment on Quality Traits of Pear

The “Laiyang” pear is a climacteric fruit with a clear peak respiration rate. During the cold storage period, the respiration rate of CK fruit first increased and then decreased, which shows a similar level at the end of storage time in comparison to beginning. A significant suppression of respiration rate was observed in 1-MCP-treated fruit compared to CK fruit. The respiratory climacteric peak of 1-MCP-treated fruit was significantly delayed by 30 days and decreased by 34.4% compared to CK fruit (Supplementary Figure S1a). A similar change was observed in ethylene production. As shown in Supplementary Figure S1b, the ethylene production in 1-MCP-treated fruit was always lower than that in the CK fruit during the storage period. The ethylene production peaks of 1-MCP-treated fruit was also delayed by 30 days and decreased by 34.3% compared to CK fruit.

In the course of the cold storage period, the firmness of the pear fruit, without treatment, decreased. As shown in Supplementary Figure S1c, the treatment with 1-MCP significantly suppressed the reduction of firmness in comparison to CK. The firmness of 1-MCP-treated fruit was 23.6 N, which was 40.6% higher than that of the CK fruit (16.8 N) at the end of storage. TA in both CK and 1-MCP treated fruit gradually increased within 90 days (Supplementary Figure S1d) and then decreased during the remaining storage time. 1-MCP treatment inhibited the decline of TA compared to CK, especially in the later storage period. The SSC of CK fruits increased to a peak value on day 30, whereas 1-MCP treatment inhibited the increase and delayed the peak to day 90. The SSC content in the 1-MCP treatment group was 7.9% lower than that in CK fruits at the end of storage (Supplementary Figure S1e). EC in the CK fruits gradually increased from 27.9% at the beginning of storage to 89.3% after 150 days of storage. The significant changing trend of EC was mitigated by 1-MCP treatment. The EC in CK fruits on day 60 of the storage period increased to 76.0%, while that value was only 54.5% in 1-MCP-treated fruits (Supplementary Figure S1f).

### Effects of 1-MCP Treatment on Aroma-Related Enzyme Activities of Pear

**FIGURE 1:**
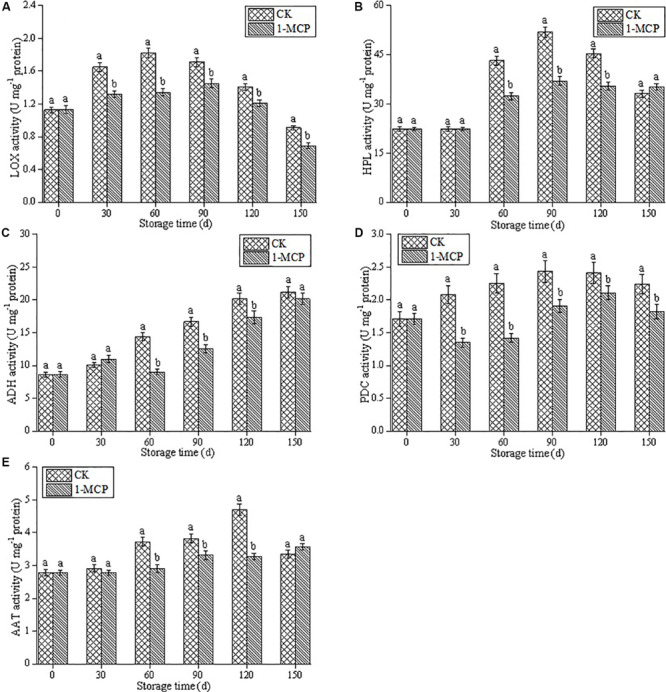
Effects of 1-MCP on LOX activity **(A)**, HPL activity **(B)**, ADH activity **(C)**, PDC activity **(D)** and AAT activity **(E)** in pear fruit during storage at 0 ± 1°C. Vertical bars represent the standard errors of the means. Means followed by different letters at the same time differ significantly at *P* < 0.05 by Duncan’s multiple range test.

Lipoxygenase activity increased significantly in the first 60 days of the cold storage period and then declined sharply in CK fruits, which exhibited a higher LOX activity than that in 1-MCP treated fruits (Figure 1A). As shown in Figures 1B,C, the activities of HPL and ADH were also inhibited by 1-MCP treatment, and significantly higher contents of HPL and ADH were found in CK fruits, except on days 30 and 150 (*P* < 0.05). Compared with the CK fruits, 1-MCP treatment also significantly inhibited PDC activity, which with a value of 18.8%, is lower than that in the CK fruits at the end of storage time (Figure 1D). The AAT activity in CK fruits gradually increased and reached the peak on day 120, whereas 1-MCP treatment significantly inhibited the increase of AAT activity, except on days 30 and 150. In particular, on day 120, the AAT activity in CK fruits was 43.6% higher than that in 1-MCP-treated fruits (Figure 1E) (*p* < 0.05).

### Effects of 1-MCP Treatment on Aroma Components of Pear

In CK fruits, 21, 27, 26, 27, 27, and 24 aroma components were respectively detected on days 0, 30, 60, 90, 120, and 150, whereas the number of aroma components detected in 1-MCP-treated fruits on days 30, 60, 90, 120, and 150 were decreased by 4, 1, 4, 3, and 3, respectively (Supplementary Table S1). Thirty aroma-related compounds were identified in the “Laiyang” pear fruit, including five alcohols, three acids, 10 esters, three ketones, three alkanes, three aldehydes, and three other aroma-related compounds in this study (Supplementary Table S2).

**FIGURE 2:**
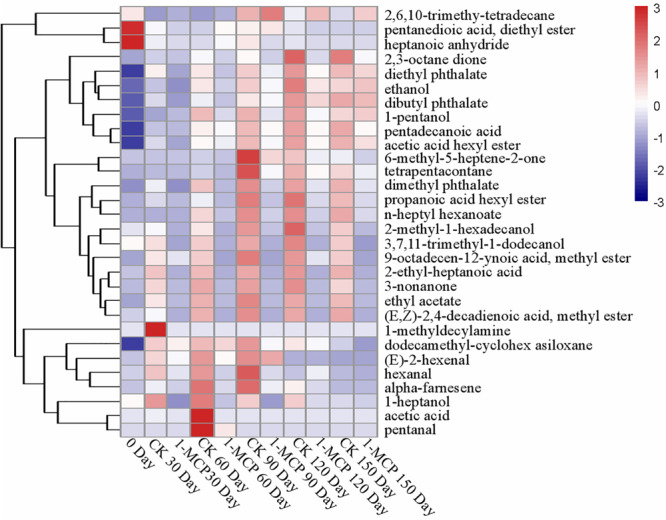
Hierarchical clustering of a heatmap depicting the aroma (Supplementary Table S2) among CK and 1-MCP. The different treatments were shown in columns, and volatile aroma compounds were shown in rows. The color box for each compound indicates the abundance of the compound. The red color annotates higher abundance while the blue color annotates the lower abundance.

As shown in Figure 2 and Supplementary Table S2, the treatment with 1-MCP significantly suppressed the aroma compounds. 2-methyl-1-hexadecanol is the most abundant alcohols in the “Laiyang” pear fruit and shows a higher content in CK fruits (*p* < 0.05). Contents of ethanol, 3,7,11-trimethyl-1-dodecanol, 1-pentanol, and 1-heptanol in the 1-MCP treatment group were also relatively lower as compared with CK fruits. Total acids displayed a fluctuation during cold storage (Supplementary Table S2). 2-ethyl-heptanoic acid accounted for the major part of these three acids and its content in CK fruits was 841% higher than that in 1-MCP-treated fruits at the end of the storage period. Total esters increased before day 90 (CK) or day 150 (1-MCP treatment group). Acetic acid hexyl ester, dimethyl phthalate, and diethyl phthalate accounted for the major part of all detected ester components and their contents in CK fruits were significantly higher than those in 1-MCP-treated fruits (*p* < 0.05) (Supplementary Table S2). Total ketones increased before day 90 and then decreased, and the contents of these ketones in CK fruits were significantly higher than those in 1-MCP-treated fruits. Three alkanes, such as dodecamethyl-cyclohexasiloxane, 2,6,10-trimethyl-Tetradecane, and tetrapentacontane were detected in “Laiyang” pear fruits. As observed for ketones, total alkanes contents in 1-MCP-treated fruits were much lower than that in CK fruits. Aldehydes including hexanal, (E)-2-hexenal, and pentanal were identified in this study. The content of (E)-2-hexenal in CK fruits first increased and then decreased during the storage period. The content of (E)-2-hexenal in CK fruits on days 30 and 60 was significantly higher than that in 1-MCP-treated fruits (*p* < 0.05) (Figure 2). The contents of pentanal, both in 1-MCP-treated and CK fruit, were only detected on day 60 during the storage time. In addition, there were three other aromas including alpha-farnesene, heptanoic acid anhydride, and 1-methyldecylamine, which were only detected at specific storage times (Figure 2).

### Effects of Ethephon Treatment on Quality Traits in Pears Treated With or Without 1-MCP, After 150 Days of Cold Storage and 7 Days of Shelf-Life

**FIGURE 3:**
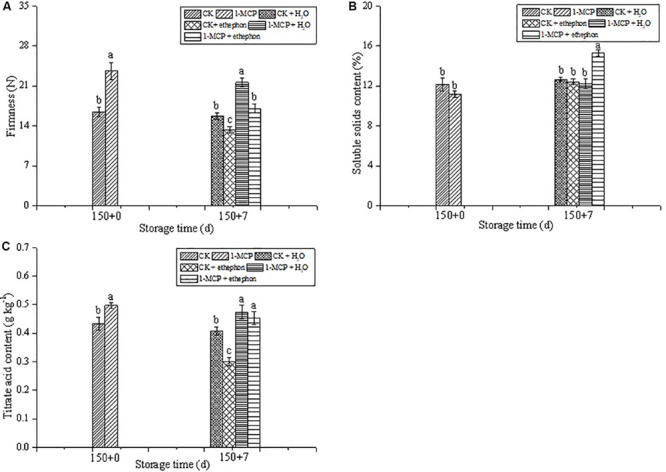
Effects of ethephon treatment on firmness **(A)**, soluble solids content **(B)**, and titrate acid content **(C)** in pear after cold storage for 150 days + 7 days of shelf-life at 20 ± 1°C. Vertical bars represent the standard errors of the means. Means followed by different letters at the same time differ significantly at *P* < 0.05 by Duncan’s multiple range test.

As shown in Figure 3A, there were no significant changes observed in the firmness of fruit not treated by ethephon during the shelf life period, while the fruit firmness significantly reduced in both CK + ethephon or 1-MCP + ethephon treatment compared with those in CK or 1-MCP-treated fruits, respectively. During the 7 days shelf-life, the SSC increased by 37.1% in the 1-MCP + ethephon treatment group, but no significant difference was found among the other three treatments (Figure 3B). The titrate acid of CK fruit dropped sharply at the shelf-life period after treatment with ethephon, whereas no significant differences were found in the fruits treated with 1-MCP + H_2_O, and 1-MCP + ethephon (Figure 3C).

### Effect of Ethephon Treatment on Aroma-Related Enzyme Activities in Pear Treated With or Without 1-MCP After 150 Days Cold Storage and 7 Days Shelf-Life

**FIGURE 4:**
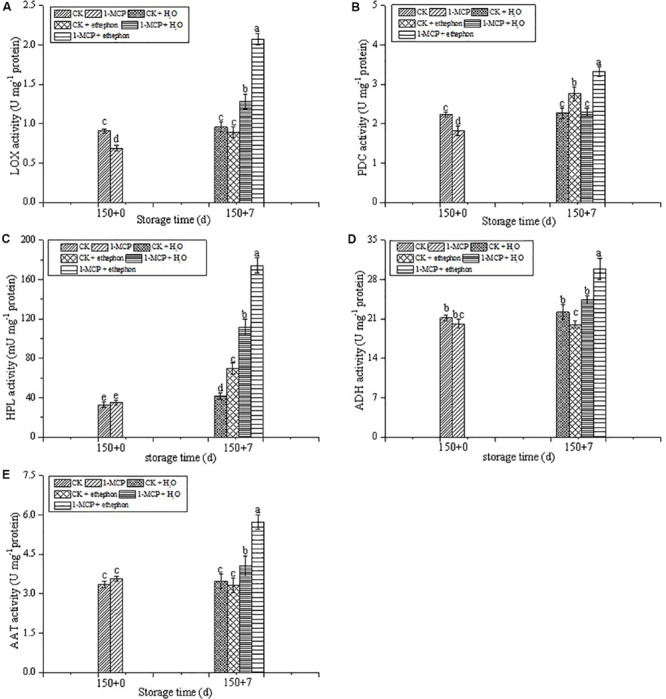
Effects of ethephon treatment on the activities of LOX **(A)**, PDC **(B)**, HPL **(C)**, ADH **(D)**, and AAT **(E)** in pear after cold storage for 150 days + 7 days of shelf-life at 20 ± 1°C. Vertical bars represent the standard errors of the means. Means followed by different letters at the same time differ significantly at *P* < 0.05 by Duncan’s multiple range test.

During the shelf-life, the LOX activity in the fruits treated with 1-MCP alone or 1-MCP + ethephon was significantly higher than that in CK fruits. In addition, the LOX activity in the 1-MCP + ethephon treatment group was 63.1% higher than that in the 1-MCP + H_2_O treatment group during the shelf-life, whereas the LOX activity in the CK + ethephon treatment group was 7.8% lower than that in the CK + H_2_O treatment group during the shelf-life (Figure 4A).

As shown in Figures 4B,C, treatment with ethephon significantly promoted the increase of PDC and HPL activities, no matter whether the fruits were treated with 1-MCP or not. The activities of PDC and HPL in the 1-MCP + ethephon treatment group were, respectively, 44.8 and 56.4% higher than those in the 1-MCP + H_2_O treatment group during the shelf-life. The PDC and HPL activities in the CK + ethephon treatment group were, respectively, 21.6 and 66.4% higher than those in the CK + H_2_O treatment group. In addition, the activity of PDC in the CK + ethephon treated fruit was higher than that in the 1-MCP + H_2_O group.

During the shelf-life, the highest ADH activity was observed in the 1-MCP + ethephon treatment group. No significant difference of ADH activity was found between the CK + H_2_O and 1-MCP + H_2_O treatment groups, whereas ADH activity in CK fruits treated with ethephon was slightly decreased (Figure 4D). The AAT activity of the 1-MCP group treated with or without ethephon was significantly increased during the shelf-life, but no significant difference was found in the CK groups (Figure 4E). In addition, the highest activity of AAT was also found in the 1-MCP + ethephon treatment group.

### Effect of Ethephon Treatment on Aroma Components of Pear Treated With or Without 1-MCP After 150 Days Cold Storage and 7 Days Shelf-Life

The total alcohol contents in the CK + H_2_O, 1-MCP + H_2_O, and 1-MCP + ethephon treatment groups were all increased during the shelf-life. The highest total alcohol content was found in the 1-MCP + ethephon treatment group, followed by the CK + H_2_O, 1-MCP + H_2_O, and CK + ethephon treatment groups. Five alcohols identified in fruits during the shelf life were also found during the cold storage period. 2-methyl-1-hexadecanol was the major alcohol in pears. 2-methyl-1-hexadecanol content was the highest, amongst all the treatment groups, in the 1-MCP + ethephon treatment group. Most notably, the contents of another four alcohols including 3,7,11-trimethyl-1-dodecanol, 1-pentanol, ethanol, and 1-heptanol in the 1-MCP + ethephon treatment group increased significantly during the shelf-life and were much higher than those in the other three treatment groups. The acids were all significantly increased in the 1-MCP + H_2_O/ethephon treatment group during the shelf-life period (Supplementary Table S3).

The total ester contents in the 1-MCP treatment group and CK treatment group, during the 150 days storage at 0 ± 1°C, were lower than that in the 1-MCP + H_2_O and 1-MCP + ethephon treatment groups in the shelf life, but higher than that in the CK + H_2_O and CK + ethephon treatment group in the shelf life (Supplementary Table S3). Acetic acid hexyl ester, dimethyl phthalate, and diethyl phthalate were the three major esters found and all increased sharply in the 1-MCP + ethephon treatment group during the shelf-life period, the contents of which were the highest in the 1-MCP + ethephon treatment group, with a value about 63.3, 80.1, and 51.7% higher than those in the 1-MCP + H_2_O, CK + ethephon and CK + H_2_O treatment groups. Total ketones in all the treatment groups showed a similar changing trend to the esters during the shelf-life, but the total content of ketones was much lower than that of esters (Supplementary Table S3).

The total content of alkanes in all the treatment groups during the shelf life was higher than that in the fruits during the 150 days storage period at 0 ± 1°C. The highest total content of alkanes found amongst all the treatment groups was in the 1-MCP + ethephon treatment group. 2,3-octane dione and 6-methyl-5-heptene-2-one were the two main alkanes identified. All three alkanes were increased during the shelf-life, except for dodecamethyl-cyclohexasiloxane, which was not detected in the CK + ethephon or 1-MCP + ethephon treatment group during the shelf-life (Supplementary Table S3).

Aldehydes were only detected in a specific treatment. As for (E)-2-hexeanl, no significant difference was found between the CK + H_2_O and CK + ethephon treatment groups. The compound of pentanal was only detected in the CK + ethephon and 1-MCP + ethephon treatment groups. In addition, alpha-farnesene and heptanoic acid anhydride were also detected in all treated fruits during the shelf-life, the content of which were also highest in the 1-MCP + ethephon treatment group (Supplementary Table S3).

### Principal Component Analysis (PCA) and Hierarchical Clustering Heatmap Analysis

**FIGURE 5:**
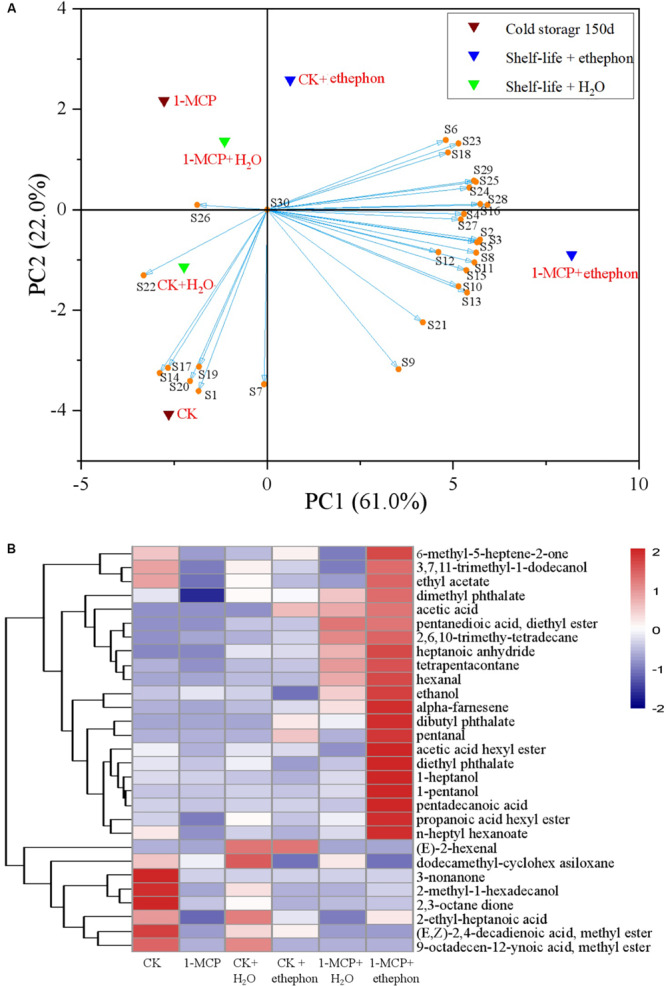
Principal component analysis (PCA) biplot **(A)** of the first two principal components (PC1 and PC2) and hierarchical cluster analysis **(B)** of the aroma compounds in different treatment pears. **(A)** Control or pear fruit treated with 1-MCP during cold storage for 150 days are indicated as CK and 1-MCP, respectively. During 7 days of shelf life, the fruit further treated with 2 mmol L^–1^ ethephon (marked as CK + ethephon or 1-MCP + ethephon) or distilled water (marked as CK + H_2_O or 1-MCP + H_2_O). Compounds represented in S from Supplementary Table S3. **(B)** Hierarchical clustering of a heatmap depicting the aroma (Table 3) among CK, 1-MCP, CK + H_2_O, 1-MCP + H_2_O, CK + ethephon and 1-MCP + ethephon treatments. The different treatments were shown in columns, and volatile aroma compounds were shown in rows. The color box for each compound indicates the abundance of the compound. The red color annotates higher abundance while the blue color annotates the lower abundance.

The effect of ethephon treatment on the aroma production in fruits during the shelf-life period (Supplementary Table S3) was also clearly depicted in the PCA plot (Figure 5A). The PCA highlighted the distribution of the samples in a hyperspace defined by two principal components (PC1: 61.0% and PC2: 22.0%), which accounted for 83.0% of the entire variability in the aroma components during the shelf life. The distinct profile of aroma components in the 1-MCP + ethephon treatment group was observed in the PCA plot (Figure 5A). CK, CK + H_2_O, 1-MCP, and 1-MCP + H_2_O treatment groups were located in the PC1 negative area of the plot, and the 1-MCP + ethephon treatment group was located in the extreme region of the PC1 positive quadrant. In addition, the CK + ethephon treatment group was also located in the PC1 positive quadrant but near the positive/negative boundary. Remarkably, the second principal component (PC2) distinguished the 1-MCP and 1-MCP + H_2_O treatment groups from the CK and CK + H_2_O treatment groups in the PC1 negative area of the plot. The 1-MCP + ethephon and the CK + ethephon treatment group in the PC1 positive area of the plot were also distinguished from each other by the PC2. The CK and 1-MCP + ethephon treatment groups were clearly discriminated from all other samples. 2-methyl-1-hexadecanol (S1), (E,Z)-2,4-decadienoic acid, methyl ester (S14), 9-octadecen-12-ynoic acid, methyl ester (S17), 3-non-anone (S19), and 2,3-octane dione (S20) contributed more to the aroma components of the CK group. On the other hand, acetic acid (S6), 2,6,10-trimethyl-tetradecane (S23), pentanedioic acid, diethyl ester (S18), heptanoic anhydride (S29), hexanal (S25), tetrapentacontane (S24), alpha-farnesene (S28), dibutyl phthalate (S16), ethanol (S4), pentanal (S27), 3,7,11-trimethyl-1-dodecanol (S2), 1-pentanol (S3), 1-heptanol (S5), pentadecanoic acid (S8), propanoic acid hexyl ester (S11), diethyl phthalate (S15), acetic acid hexyl ester (S10), n-heptyl hexanoate (S13), 6-methyl-5-heptene-2-one (S21), and ethyl acetate (S9) contributed more to the aroma components of the 1-MCP + ethephon treatment group.

The specific content of aromas was furthermore graphically reordered with a hierarchical clustering heatmap (Figure 5B) that showed a gradual and linear shift of aroma components from the CK to 1-MCP + ethephon treatment group. The cluster of aromas with lower content in 1-MCP was significantly increased in the 1-MCP + ethephon treatment group, while, on the contrary, the aroma with a higher content in CK resulted in a strong decrease in the CK + ethephon treatment group.

## Discussion

1-MCP as a competitive inhibitor, binding irreversibly to the ethylene receptor, blocks ethylene action and influences ethylene biosynthesis through feedback inhibition in a broad range of horticultural crops. Therefore, post-harvest application of 1-MCP is a common and effective technology for the quality maintenance of various fruits and vegetables (Cai et al., 2006; Façanha et al., 2019; Massolo et al., 2019). Previous research on “d” Anjou’ pears (Argenta et al., 2003), “William’s” pears (Xie et al., 2014), “Red Clapp’s” pears (Calvo and Sozzi, 2004), and “Packham’s Triumph” pears (Moya-León et al., 2006) showed that 1-MCP treatment had tremendous potential for maintaining the quality of pear fruits and significantly inhibited the development of physiological disorders during storage. Our previous results also demonstrated that 0.5 μl L^–1^ 1-MCP treatment effectively prevented fruit core browning and maintained Vc contents and flesh color of the “Laiyang” pear fruit (Li F. et al., 2013). On this basis, we further explored its effects on fruit quality and aroma components in the present study. The results demonstrated that treatment with 0.5 μl L^–1^ 1-MCP significantly delayed fruit senescence, as shown in Supplementary Figure S1. Although the firmness and titratable acidity were not affected by 1-MCP within 90 days, 1-MCP treatment significantly inhibited the decline of firmness and titratable acidity at the later stage of storage. In the study, we also found that the accumulation of aroma volatile compounds in “Laiyang” pear fruits was inhibited by 1-MCP during the cold storage. Previous research found that volatile compounds were produced through several biosynthetic pathways, and fatty acid metabolism was a key pathway for aroma volatile biosynthesis in most fruits, which comprises four key enzymes, including LOX, HPL, ADH, and AAT (Bartley et al., 1985; Dudareva et al., 2013). LOX and HPL are essential for the formation of aldehydes, which are reduced to alcohols in ADH-catalyzed reactions (Defilippi et al., 2009; Dudareva et al., 2013). AAT catalyzes the biosynthesis of esters and a significant correlation between AAT activity and ester formation has been reported in pears (*Pyrus ussuriensis* Maxim) (Li G. et al., 2013). In the study, the contents of aldehydes increased before 90 days and then decreased. Analysis of the activities of LOX and HPL found that the change trend of the enzymes was similar to that of the aldehydes content. Production of total esters aroma compounds are very low in fruits treated with 1-MCP in comparison to CK. According to Rizzolo et al. (2005) ethyl acetate was identified as an important volatile in the “Conference” pear aroma. We found that ethyl acetate, acetic acid hexyl ester, dimethyl phthalate, diethyl phthalate and (E,Z)-2,4-Decadienoic acid, and methyl ester accounted for most of the ester aroma of “Laiyang” pears. These ester aroma compounds were also detected in “Conference,” “Alexander Lucas,” and “Nanguoli” pears (Li G. et al., 2013; Hendges et al., 2018). In addition, dimethyl phthalate and diethyl phthalate were also detected in “Laiyang” pears in our study, which have not been reported in other pear varieties, but which have been reported in other fruits such as longans and peaches (Chen et al., 2010; Xu et al., 2012). The highest content of esters was found in CK fruit after being stored for 120 days, which was closely related the activity of AAT (Figure 1E). Particularly, 1-MCP treatment significantly suppressed the increase of ethyl acetate, propanoic acid hexyl ester, dimethyl phthalate, n-heptyl hexanoate, (E,Z)-2,4-Decadienoic acid methyl ester, and 9-octadecen-12-ynoic acid methyl ester. In addition, though the activities of ADH, HPL, and AAT showed no significant difference between CK and 1-MCP on day 30, the aroma compounds of alcohols, aldehydes and esters were higher in CK. The contents of 1-pentanol, ethanol, n-heptyl hexanoate, dibutyl phthalate, and dodecamethyl-cyclohexasiloxane showed no significant difference according to the activities of ADH, HPL, and AAT. Previous research also noted that 1-MCP treatment reduced the characteristic aroma in “Nanguo” Pears (Dong et al., 2010). A lower production of alcohols, aldehydes, and esters was found in d’Anjou pears treated with 1-MCP (Argenta et al., 2003). Hendges et al. (2018) found lower levels of 1-butanol, butyl, and hexyl acetates in “Conference” pears which were harvested from the first maturity stage and treated with 1-MCP before cold storage. Therefore, although 1-MCP could delay the post-harvest senescence of pear fruits, its negative effects on the formation of aroma compounds should not be overlooked. How to maintain the physiological quality of the fruit and how to reduce the loss of aroma has become a problem that needs to be solved.

The mechanisms of 1-MCP inhibition of aroma formation were reportedly due to the suppression of the expression of genes involved in volatiles synthesis, including *LOX*, *HPL*, *ADH*, and *AAT* in fruits (Li G. et al., 2016; Cai et al., 2018). In our study, the significant depression of the activities of aroma-related enzymes (LOX, HPL, ADH, PDC, and AAT) was found in the 1-MCP treatment group. Despite the fact that an inhibitory effect of 1-MCP on volatile aroma has been discovered in fruit, the way to restore aroma has seldomly been reported. It is well known that ethylene plays an important role in the ripening of climacteric fruits and initiating and coordinating diverse processes including the aroma formation (Torregrosa et al., 2020). Günther et al. (2015) also reported that a reduction in the rate of ethylene production during refrigerated storage resulted in a decrease in the production of volatile aroma compounds. Ethephon influences several metabolic pathways of climacteric fruits and plays an important role in the formation of aroma compounds (Xu et al., 2015; Li Y. et al., 2016; Wang et al., 2016). Schaffer et al. (2007) showed that a transgenic line of apple with no detection of ethylene had very low aroma levels. We explored the role of ethylene in aroma recovery by measuring the effect of ethephon on the aroma component and content of pears under different treatments. Combined with the detection of aroma related enzymes, we found that activities of aroma-related enzymes (LOX, HPL, ADH, PDC, AAT) in 1-MCP + ethephon, improved significantly compared to other treatments. Moreover, we found that the activity of PDC in CK + ethephon was higher than that in 1-MCP + H_2_O. Analysis of aldehydes showed that (E)-2-hexenal and pentanal were higher in CK + ethephon. In addition, the ester aroma compounds such as acetic acid hexyl ester, propanoic acid hexyl ester, dimethyl phthalate, n-heptyl hexanoate, diethyl phthalate, dibutyl phthalate, pentanedioic acid, and diethyl ester (Figure 5B) improved significantly in 1-MCP + ethephon fruits. We analyzed the results and found that the enhanced ester aroma compounds may be related to the increase of AAT activity, which was similar to the results reported by Li G. et al. (2013). Moreover, Li Y. et al. (2016) reported that the level of acetate, hexanoate, and hexyl esters and the activities of LOX, ADH, and AAT in sweet melon were significantly increased by ethephon. The hexanal and pentanal are two main aldehydes which increased 12.16 and 5.09 times, respectively, in 1-MCP + ethephon treated fruits compared with 1-MCP treated fruits. These results suggested that the additional application of ethephon during shelf-life was a promising way to allow recovery of aroma components after long-term cold storage of “Laiyang” pears.

In conclusion, compared with the storage at 0 ± 1°C without any treatment, 1-MCP treatment more effectively controlled ripening and senescence of Laiyang pear fruits under cold storage conditions. However, the activities of aroma-related enzymes, such as LOX, PDC, HPL, ADH, and AAT, as well as the important aroma volatiles, such as esters and ketone, were negatively influenced by 1-MCP treatment during the cold storage period. During the shelf-life period after 150 days of cold storage, the application of ethephon could significantly increase the activities of aroma-related enzymes (LOX, PDC, HPL, ADH, and AAT) and the aroma components. Moreover, compared with the CK group, without being treated with 1-MCP or ethephon, 1-MCP + ethephon treatment had no effect on fruit firmness, but it increased the contents of SSC and TA in fruits during the shelf-life period. Therefore, the application of ethephon during shelf-life has the potential for commercial application to allow recovery of aroma components after long-term cold storage of “Laiyang” pears.

## Data Availability Statement

All datasets generated or analyzed for this study are included in the article.

## Author Contributions

XZ and FL conceived and designed the research. PS drafted the manuscript and analyzed all data. DM, JZ, WA, JL, and ZL carried out most of the experiments together, with close supervision from XZ, XL, and YG. ZS and YS performed the quality indexes analysis. All authors read and approved the manuscript.

## Conflict of Interest

The authors declare that the research was conducted in the absence of any commercial or financial relationships that could be construed as a potential conflict of interest.
