# Supplementary material for: The Synergism of 1-Methylcyclopropene and Ethephon Preserves Quality of “Laiyang” Pears With Recovery of Aroma Formation After Long-Term Cold Storage
Source: Front Plant Sci. 2020 May 25;11:490. doi: 10.3389/fpls.2020.00490 (PMC7261920; doi:10.3389/fpls.2020.00490)
Supplement: Supplementary file 1 [file Data_Sheet_1.pdf]

Supplementary Material

Table S1 Effects of 1-MCP on aroma quantities of pear during storage at 0 ± 1 °C.

Category	0 d	30 d		60 d		90 d		120 d		150 d	
		CK	1-MCP	CK	1-MCP	CK	1-MCP	CK	1-MCP	CK	1-MCP
Alcohol	4	5	5	5	5	5	5	5	4	5	5
Acid	3	3	3	3	3	2	2	2	2	2	2
Ester	7	9	7	9	8	10	8	10	7	8	7
Ketone	1	3	2	3	2	3	2	3	5	3	2
Alkane	2	2	3	2	3	3	3	3	4	3	3
Aldehyde	2	2	2	3	3	2	2	2	1	2	1
Alpha-Farnesenes	1	1	1	1	1	1	1	1	1	1	1
Anhydride	1	1	0	0	1	1	0	1	0	0	0
Amine	0	1	0	0	0	0	0	0	0	0	0
Total	21	27	23	26	25	27	23	27	24	24	21

Table S2 Effects of 1-MCP on the changes of aroma-related volatiles in pear during storage at 0 ± 1 °C.[a]

Category	Name	0d	30d		60d		90d		120d		150d	
			CK	1-MCP	CK	1-MCP	CK	1-MCP	CK	1-MCP	CK	1-MCP
Alcohol	2-methyl-1-hexadecanol	10.74±0.12	14.37±0.12a	3.72±0.13b	19.54±0.29a	3.98±0.24b	33.03±3.15a	6.50±0.32b	43.23±2.45a	5.96±0.07b	23.55±1.34a	4.48±0.29b
	3,7,11-trimethyl-1-dodecanol	11.86±0.04	15.36±0.31a	2.49±0.05b	17.34±0.13a	3.78±0.09b	21.15±0.14a	4.11±0.06b	25.41±0.13a	3.88±0.05b	17.22±0.11a	1.57±0.15b
	1-pentanol	-	0.30±0.04a	0.41±0.06a	0.99±0.07a	0.53±0.06b	1.06±0.24a	0.58±0.08b	1.12±0.16a	0.68±0.08b	0.89±0.09a	0.92±0.09a
	ethanol	5.89±0.52	6.34±0.72a	6.63±0.42a	8.72±0.81a	7.65±0.55b	9.24±073a	8.21±0.72b	10.84±0.95a	8.72±0.67b	9.12±0.87a	9.54±0.79b
	1-heptanol	3.54±0.05	5.82±0.21a	1.18±0.03b	6.53±0.02a	2.26±0.06b	4.62±0.04a	1.53±0.04b	4.70±0.05a	2.61±0.09b	2.62±0.08a	2.47±0.07a
Total		32.03	43.19a	14.43b	53.12a	18.20b	69.10a	20.93b	85.31a	21.85b	53.40a	18.98b
Acid	acetic acid	0.25±0.03	0.63±0.08a	0.28±0.05b	9.51±1.12a	0.31±0.02b	-	-	-	-	-	-
	2-ethyl-heptanoic acid	4.73±0.52	17.82±2.11a	1.55±0.17b	19.21±1.89a	1.86±0.19b	21.62±2.17a	1.88±0.23b	23.35±2.15a	2.19±0.78b	19.76±1.85a	2.35±0.12a
	pentadecanoic acid	1.51±0.16	7.11±0.35a	6.52±0.68b	9.92±0.68a	9.12±0.79a	12.53±1.17a	9.45±0.79b	13.71±1.24a	9.72±0.97b	13.25±1.44a	9.64±1.52b
Total		6.49	25.56a	8.35b	38.64a	11.30b	34.15a	11.33b	37.06a	11.91b	33.01a	11.99b
Ester	ethyl acetate	-	17.53±1.35a	3.16±0.31b	27.93±2.14	3.55±0.41b	35.66±2.13a	3.67±0.43b	30.21±1.52a	3.72±0.34b	26.55±2.11a	3.94±0.42b
	acetic acid, hexyl ester	10.54±1.56	41.68±2.87a	31.56±3.82b	50.76±4.14a	45.57±2.98b	65.28±0.34a	48.82±0.53b	72.45±0.56a	51.12±0.34b	68.22±5.2a	56.71±3.4b
	propanoic acid hexyl ester	1.54±0.17	7.16±0.72a	1.78±0.18b	9.13±0.94a	2.78±0.35b	31.73±2.45a	4.22±0.28b	33.28±2.97a	6.26±0.72b	22.87±2.12a	7.07±0.73b
	dimethyl phthalate	21.30±2.11	75.20±1.4a	23.35±1.72b	122.2±4.18a	45.36±4.21b	152.81±4.72a	60.14±2.87b	145.53±5.12a	70.37±5.21b	133.21±8.14a	72.27±7.12b
	n-heptyl hexanoate	-	-	-	9.79±0.83a	1.54±0.22b	16.78±1.22a	2.18±0.24b	16.97±1.79a	2.66±0.32b	14.26±1.42a	3.11±0.32b
	(E,Z)-2,4-Decadienoic acid, methyl ester	6.47±0.83	13.36±2.05a	-	33.52±3.07a	-	41.17±0.06a	-	35.66±0.38a	-	35.21±0.37a	-
	diethyl phthalate	104.4±6.72	198.2±7.27a	147.8±5.72b	201.7±7.21a	163.6±9.19b	217.9±17.32a	183.2±10.23b	243.1±12.31a	191.1±13.15b	223.7±11.23a	212.2±12.16b
	dibutyl phthalate	2.12±0.15	4.51±0.53a	4.34±0.21a	4.82±0.38a	4.13±0.54b	5.71±0.62a	5.12±1.73b	7.38±0.57a	6.11±0.74b	7.11±0.82a	6.72±1.61b
	9-octadecen-12-ynoic acid, methyl ester	-	9.71±0.08a	5.05±0.21b	12.90±0.38a	-	20.72±1.91a	-	17.76±0.24a	5.82±0.13b	12.56±0.16a	-

	pentanedioic acid, diethyl ester	1.33±0.15	0.20±0.01	-	-	0.26±0.03	0.28±0.04a	0.33±0.17b	0.11±0.02a	0.04±0.00b	-	-
Total		145.58	367.56a	216.04b	472.76a	266.81b	588.05a	307.65b	602.49a	337.22b	543.69a	362.02b
Ketone	3-nonanone	-	0.30±0.04	-	0.41±0.05	-	0.45±0.03	-	0.48±0.05	-	0.30±0.04	-
	2,3-octane dione	-	0.63±0.05a	0.46±0.08b	1.17±0.09a	0.55±0.05b	1.35±0.15a	0.67±0.05b	4.22±0.42	0.68±0.08b	3.71±0.42a	1.23±0.11b
	6-Methyl-5-heptene-2-one	0.54	0.73±0.08a	0.62±0.07b	0.93±0.09a	0.71±0.08b	16.34±3.82a	6.53±0.48b	7.82±0.07a	2.77±0.05a	3.13±0.09a	0.86±0.07b
Total		0.54	1.66a	1.08b	2.51a	1.26b	18.14a	7.20b	12.52a	3.45b	7.14a	2.09b
Alkane	dodecamethyl-cyclohexasiloxane	-	2.80±0.15a	2.40±0.12a	3.04±0.26a	2.66±0.03a	3.52±0.12a	2.12±0.11b	2.53±0.16a	2.15±0.17a	1.67±0.08a	1.03±0.02b
	2,6,10-trimethyl-tetradecane	4.78±0.32	-	0.82±0.07a	-	0.95±0.26a	6.97±0.31b	9.13±0.21a	3.02±0.22b	6.43±0.36a	2.49±0.08b	5.67±0.11a
	tetrapentacontane	0.69±0.07	1.58±0.18a	0.88±0.13b	2.78±0.21a	1.46±0.13b	23.74±2.13a	6.72±0.21b	14.03±0.45a	7.23±0.15b	10.79±1.16a	5.47±0.48b
Total		5.47	4.38a	4.10a	6.18a	5.07b	34.23a	17.97b	19.58a	15.81b	14.95a	12.17b
Aldehyde	hexanal	1.30±0.15	4.60±0.37a	1.86±0.22b	6.08±0.71a	1.23±0.21b	8.36±0.57a	1.37±0.16b	0.78±0.04a	2.23±0.07b	0.44±0.02a	0.40±0.03a
	(E)-2-hexenal	1.15±0.14	4.85±0.24a	2.85±0.21b	7.28±0.38a	3.36±0.14b	7.53±0.37a	6.51±0.53a	0.50±0.06a	0.48±0.05a	0.13±0.04a	-
	pentanal	-	-	-	0.14±0.02a	0.03±0.00b	-	-	-	-	-	-
Total		2.45	9.45a	4.71b	13.50a	4.62b	15.89a	7.88b	1.28a	2.61b	0.57a	0.40b
Alpha-farnesene	alpha-farnesene	1.75±0.16	4.41±0.21a	2.63±0.08b	16.34±1.92a	3.37±0.14b	17.25±1.62a	4.56±0.05b	6.74±0.21a	2.95±0.06b	1.08±0.04a	0.91±0.03a
Anhydride	heptanoic anhydride	2.28±0.31	0.24±0.05	-	-	0.32±0.04	0.55±0.02	-	0.16±0.00	-	-	-
Amine	1-methyldecylamine	-	0.02±0.00	-	-	-	-	-	-	-	-	-
Total		4.03	4.67a	2.63b	16.34a	3.70b	17.80a	4.56b	6.90a	2.95b	1.08a	0.91a

[a] Means (Unit: µg/g) with different letters in a column for each parameter at the same time differ significantly at p=0.05 according to Duncan's multiple range tests. Data are the means ± standard error (SE). "-" means not detected

Table S3 Effects of ethephon on the changes of aroma-related volatiles in pear after cold storage for 150 d + 7 d of shelf-life at 20 ± 1 °C.[b]

Number	Category	Name	150d		150+7d			
			CK	1-MCP	CK+H_2O	CK+ethephon	1-MCP+H_2O	1-MCP+ethephon
S1	Alcohol	2-methyl-1-hexadecanol	23.55±1.34a	4.48±0.29e	11.92±0.07c	5.27±0.11d	5.41±0.23d	17.24±0.31b
S2		3,7,11-trimethyl-1-dodecanol	17.22±0.11b	1.57±0.15e	11.28±0.14c	7.01±0.11d	1.68±0.13e	21.28±0.32a
S3		1-pentanol	0.89±0.09b	0.92±0.09b	1.06±0.15b	0.76±0.08c	1.18±0.21b	7.26±0.54a
S4		ethanol	9.12±0.87c	9.54±0.79c	9.24±0.38c	7.88±0.27d	10.69±0.51b	13.17±0.16a
S5		1-heptanol	2.62±0.08b	2.47±0.07b	2.11±0.06c	1.76±0.08b	2.55±0.04c	9.26±0.24a
	Total		53.40b	18.98e	35.61c	22.68d	21.51d	68.21a
S6	Acid	acetic acid	-	-	-	8.70±0.61c	9.51±0.72b	12.37±1.27a
S7		2-ethyl-heptanoic acid	19.76±1.85a	2.35±0.12e	21.62±1.38a	10.22±0.62c	4.21±1.83d	13.92±1.58b
S8		pentadecanoic acid	13.25±1.44b	9.64±1.52c	12.53±1.26b	12.48±1.42b	9.92±0.57c	81.18±3.29a
	Total		33.01b	11.99d	34.15b	31.40b	23.64c	107.47a
S9	Ester	ethyl acetate	26.55±2.11b	3.94±0.42f	17.34±1.22c	10.61±0.35d	7.93±2.21e	32.72±3.11a
S10		acetic acid hexyl ester	68.22±5.2b	56.71±3.4c	65.28±5.28b	63.93±3.61b	50.76±4.21d	117.63±6.11a
S11		propanoic acid hexyl ester	22.87±2.12c	7.07±0.73d	31.73±2.18b	21.14±2.14c	39.13±2.11b	74.84±5.21a
S12		dimethyl phthalate	133.21±8.14d	72.27±7.12e	142.81±10.11c	140.55±8.14c	163.80±5.33b	194.25±7.72a
S13		n-heptyl hexanoate	14.26±1.42b	3.11±0.32f	8.27±0.59c	6.18±0.44e	9.79±0.71d	33.17±2.11a
S14		(E,Z)-2,4-decadienoic acid, methyl ester	35.21±0.37a	-	15.92±0.56b	12.23±1.33c	-	-
S15		diethyl phthalate	223.7±11.23b	212.20±12.16c	227.91±5.81b	191.98±6.72d	211.72±8,82c	345.68±10.38a
S16		dibutyl phthalate	7.11±0.82d	6.72±1.61d	5.71±0.45e	35.79±3.76b	44.82±0.54c	89.49±4.96a
S17		9-octadecen-12-ynoic acid, methyl ester	12.56±0.06a	-	10.57±0.04a	-	-	-
S18		pentanedioic acid, diethyl ester	-	-	1.38±0.14b	1.41±013b	7.26±0.62a	7.28±0.32a
	Total		543.69b	362.02d	526.92b	483.82c	535.21b	895.06a
S19	Ketone	3-nonanone	0.30±0.04a	-	-	-	-	-
S20		2,3-octane dione	3.71±0.42a	1.23±0.11c	1.73±0.25b	1.07±0.12c	1.17±0.22c	1.24±0.36c
S21		6-methyl-5-heptene-2-one	3.13±0.09a	0.86±0.07b	1.32±0.04c	2.46±0.05b	0.35±0.05c	5.23±0.33a
	Total		7.14a	2.19f	3.05d	3.53c	1.52e	6.47b
S22	Alkanes	dodecamethyl-cyclohexasiloxane	1.67±0.08b	1.03±0.02d	2.72±0.08a	-	1.33±0.02c	-
S23		2,6,10-trimethyl-tetradecane	2.49±0.08e	5.67±0.11d	9.47±0.62d	15.31±0.55c	60.21±1.23b	71.01±5.11a
S24		tetrapentacontane	10.79±1.16c	5.47±0.48d	11.74±2.71c	12.52±2.11c	37.52±2.61b	48.84±3.71a

	Total		14.95f	12.17e	23.93d	27.83c	99.06b	119.85a
S25	Aldehyde	hexanal	0.44±0.02d	0.40±0.03d	0.88±0.06c	0.92±0.07c	3.80±11.41b	5.79±0.24a
S26		（E)-2-hexenal	0.13±0.04b	-	7.53±0.62a	7.56±0.32a	-	-
S27		pentanal	-	-	-	2.39±0.15b	-	5.09±0.61a
	Total		0.57c	0.40d	8.41b	10.87a	3.80d	10.88a
S28	Alpha-farnesene	alpha-farnesene	1.08±0.04d	0.91±0.03d	1.25±0.21d	1.83±0.12c	3.40±0.14b	7.43±0.77a
S29	Anhydride	heptanoic anhydride	-	-	2.31±0.23c	1.93±0.11d	5.22±0.11b	8.33±0.43a
S30	Amine	1-methyldecylamine	-	-	-	-	-	-
	Total		1.08d	0.91d	3.56c	3.76c	8.62b	15.76a

[b] Means (Unit: μg/g) with different letters in a row for each parameter differ significantly at p=0.05 according to Duncan's multiple range tests. Data are the means ± standard error (SE). "-" means not detected. Numbers correspond to those in Fig. 5a.

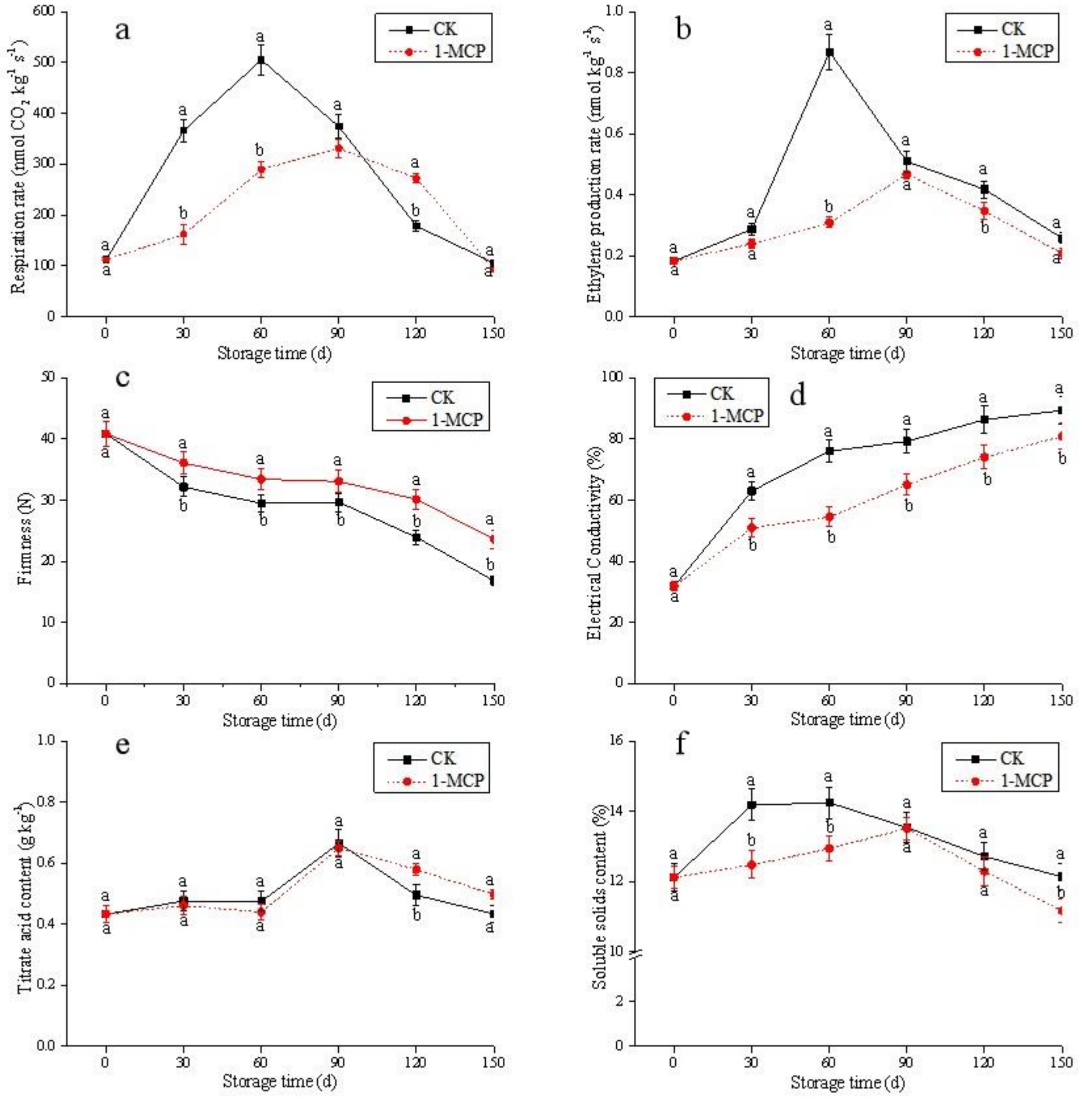

Figure S1 Effects of 1-MCP on respiration rate (a), ethylene production rate (b), firmness (c), electrical conductivity (d), titrate acid content (e) and soluble solids content (f) in pear fruit during storage at 0 ± 1 °C. Vertical bars represent the standard errors of the means.
